# Health care use and costs at the end of life: a comparison of elderly Australian decedents with and without a cancer history

**DOI:** 10.1186/s12904-017-0213-0

**Published:** 2017-06-21

**Authors:** Rebecca Reeve, Preeyaporn Srasuebkul, Julia M. Langton, Marion Haas, Rosalie Viney, Sallie-Anne Pearson, On behalf of the EOL-CC study authors

**Affiliations:** 1 Centre for Health Economics Research & Evaluation, University of Technology Sydney, PO Box 123 Broadway, Sydney, NSW 2007 Australia; 2 Faculty of Pharmacy, The University of Sydney, Sydney, NSW 2006 Australia; 3 Centre for Big Data Research in Health, Faculty of Medicine, UNSW Australia, Sydney, Australia; 4 Centre for Social Impact, UNSW Australia, Sydney, NSW 2000 Australia; 5 Centre for Health Services and Policy Research, The University of British Columbia, Vancouver, BC V6T 1Z3 Canada; 6 Department of Developmental Disability Neuropsychiatry, Faculty of Medicine, UNSW Australia, Sydney, NSW 2052 Australia

**Keywords:** End-of-life care, Terminal care, Neoplasm, Veterans health, Health care utilisation, Health care costs

## Abstract

**Background:**

There is limited population-level research on end-of-life care in Australia that considers health care use and costs across hospital and community sectors. The aim of this study was to quantify health care use and costs in the last 6 months of life in a cohort of elderly Australian decedents and to examine the factors associated with end-of-life resource use and costs.

**Methods:**

A retrospective cohort study using routinely collected health data from Australian Government Department of Veterans’ Affairs clients. The study included two cohorts of elderly Australians who died between 2005 and 2009; one cohort with a recorded cancer diagnosis and a comparison cohort with no evidence of a cancer history. We examined hospitalisations, emergency department (ED) visits, prescription drugs, clinician visits, pathology, and procedures and associated costs in the last 6 months of life. We used negative binominal regression to explore factors associated with health service use and costs.

**Results:**

The cancer cohort had significantly higher rates of health service use and 27% higher total health care costs than the comparison cohort; in both cohorts, costs were driven primarily by hospitalisations. Older age was associated with lower costs and those who died in residential aged care incurred half the costs of those who died in hospital.

**Conclusions:**

The results suggest differences in end-of-life care pathways dependent on patient factors, with younger, community-dwelling patients and those with a history of cancer incurring significantly greater costs. There is a need to examine whether the investment in end-of-life care meets patient and societal needs.

**Electronic supplementary material:**

The online version of this article (doi:10.1186/s12904-017-0213-0) contains supplementary material, which is available to authorized users.

## Background

Elderly populations continue to grow, with estimates that in Organisation for Economic Co-operation and Development (OECD) countries one in five people will be 65 years and older by 2030 [1] and, in developed countries, at least half of the population over 65 have more than one chronic condition [2–5]. As populations become sicker, they have increasing health care needs and it is not surprising that per capita health care costs increase with age [4]. However, the health care costs associated with ageing are low compared with costs incurred in the 6–12 months prior to death; some estimates show that per capita health care costs are up to four times higher in those at the end of life compared with age-matched persons who are not at life’s end. For example, Neuman et al. [4] found that in the USA, average Medicare per capita spending for beneficiaries age 96 ($US16,145) was more than double that for beneficiaries age 70 ($US7,566) [4, 6].

There is a growing literature dedicated to understanding patterns of health care use and costs at the end of life, much of which has been conducted using routinely collected health care data [6–9]. The research highlights the extensive array of health care services delivered at the end of life and the high costs associated with care. However, significant variability is reported in end-of-life health care and costs which is dependent on a range of patient and health system factors; one of the most consistently important factors is age at death [8]. Several studies show that the chance of receiving intensive or life-sustaining treatments such as chemotherapy, emergency department (ED) visits, and admission to intensive care units and hospitals decreases with age [10–16]. As such, it is not surprising that corresponding health care costs decrease steadily with age, particularly in those aged 85 years and older [4, 14, 15, 17, 18]. To date, the majority of research on end-of-life care has been undertaken in Europe or North America. Given the complexities and jurisdiction-specific features of end-of-life care for elderly patients, there is a need to conduct studies that account for local health system characteristics such as the relationship between primary and hospital-based care, level of subsidisation of services and the availability of hospice and palliative care services.

The aim of this study is to quantify health care use and costs in the last 6 months of life in the Australian setting and to examine the factors associated with end-of-life care in an elderly decedent population. We compare total health care use and costs in two elderly decedent cohorts; a cohort with a history of cancer and a cohort without a cancer history. We focused on comparing cohorts with and without a previous cancer diagnosis as cancer is the leading cause of death in Australia and other similar countries and cancer patients represent a significant proportion of patients receiving end-of-life care [19, 20]. Additionally, patterns of end-of-life cancer care are more extensively studied and understood than other terminal diseases which provides an important context within which to interpret our findings [21].

## Methods

### Setting

The Australian Government Department of Veterans’ Affairs (DVA) funds the health care of eligible veterans, war widows, war widowers and their dependents. DVA clients have access to the universal health care arrangements provided to Australian permanent residents and citizens plus additional DVA-approved services and pharmaceutical items not subsidised for the general population; the types of services subsidised depends on the level of entitlement.

### Study design and population

This study used data from the cancer and comparison cohorts developed for the End of Life in Cancer Care (EOL-CC) study, the details of which have been published elsewhere [22]. Briefly, this is a retrospective study of two decedent cohorts of Australian DVA clients with full health care entitlements during the last 12 months of life to ensure near-complete capture of health-related resource use and costs. Clients were eligible for the study if they: resided in New South Wales (Australia’s most populous state) in the 18 months prior to death; were aged 65 years or older at death; died between June 30 2005 and December 31 2009; and received at least one health service in the last 12 months of life. The cancer cohort had a notifiable cancer diagnosis recorded in the NSW Central Cancer Registry between 1994 and 2009. The comparison cohort comprised the remaining eligible clients with no evidence of a cancer notification or any cancer related health service use or cancer medicine; for more information, see our study protocol [22].

### Data sources and linkage

The data infrastructure comprises DVA data holdings linked with NSW Ministry of Health data collections. Data were linked by a third party under the custodianship of the DVA, the NSW Ministry of Health and the Cancer Institute NSW, using best practice, probabilistic, privacy preserving protocols [23]. The linked datasets capture information on DVA client characteristics, residence in an aged care facility, cancer notification history and cause of death. However, we did not present information on cause of death for the two cohorts because information was only available to 2007. For those who died in the period 2005–2007, the most common causes of death in the cancer cohort were cancer (59%) and diseases of the circulatory system (21%). For the comparison cohort, the most common causes of death were diseases of the circulatory system (48%), lung disease (7%) and dementia (9%); for more information see our study protocol [23].

The linked datasets also include information about all subsidised health services including hospital admissions, emergency department (ED) presentations and dispensed prescription medicines. Clinical consultations, pathology services and procedures; procedures include diagnostic services (e.g., ultrasound, CT, MRI), therapeutic services (e.g., radiotherapy) and surgery are also captured; unit costs were available for all these services, while costs for the hospital admissions and ED presentations were calculated per admission or presentation using the methodology described in the NSW Costs of Care Standards Report 2009/10. Full details of the costing methods are described elsewhere [22].

### Statistical analyses

Outcomes were health service use and associated costs in the last 6 months of life based on six constructed ‘months’ consisting of 30 days each; all individuals are observed for exactly 180 days to the date of death.

We calculated person-level mean (with 95% confidence interval (CIs)) and median (with inter quartile range (IQR)) service use and costs for the entire six-month period, and by month until death.

We allocated unit costs to each item of resource use to calculate mean (95% CI) and median (IQR) costs in 2009/10 Australian dollars. Total costs were calculated as the sum of costs of health services (excluding any services received during a hospitalisation which are captured in hospital costs), pharmacy, ED and hospital costs (excluding the pharmacy component of hospital costs for private hospital patients which is captured in the prescribing database); more details are reported elsewhere [22, 24].

#### Factors associated with resource use and costs

We used negative binomial regression to determine factors associated with health service use and costs. The cost data can be thought of as counts (of dollars and cents). Cost data are non-negative and skewed so count data models can be applied to these data, and in our case this makes sense because it is consistent with the method used for utilization [25]. Factors included: age, sex, comorbidity burden, location of residence (remoteness, areas of socioeconomic disadvantage), year of death, residence in an aged care facility and place of death. These variables were selected based on common factors found to predict patterns of health service use as death approaches [22]. Comorbidity burden was estimated in the periods prior to the last 6 months of life using hospitalisation codes (Charlson index [26]) and prescription dispensing history (RxRisk [27]); while the Charlson is likely to under ascertain morbidity burden it is more likely to capture people with severe morbidity, e.g. those needing admission to hospital for the condition. Two indices were used to capture a more complete morbidity history [22, 28].

Separate models were estimated for utilisation and costs of each type of health service (i.e., clinician visits, pathology and procedures; prescription medicines dispensed; ED visits and hospitalisations) and total health care costs. The strength of associations was represented by adjusted incident rate ratios with 95% CIs, and two-tailed *p* < 0.05 were used as a criterion for statistical significance.

We used SAS version 9.3 (SAS Institute) for data manipulation and performed statistical analyses using STATA version 12 (StataCorp).

## Results

### Cohort characteristics [Table 1]

A total of 9862 decedents met the eligibility criteria for the cancer cohort and 15,483 for the comparison cohort. The cohorts were similar in relation to age, socioeconomic status and geographical location of residence. Comorbidity burden (based on hospitalisations) was higher in the cancer cohort; 17% of cancer decedents versus 10% of the comparison cohort had a comorbidity score of three or more. However, comorbidity burden was similar for both cohorts when calculated based on prescription medicines dispensed [Table 1].

**Table 1:** Cohort characteristics

	Cancer cohort (*N* = 9862)	Comparison cohort (*N* = 15,483)
	*n* (%)	*n* (%)
Sex		
Female	3116 (31.6)	7521 (48.9)
Male	6746 (68.4)	7962 (51.4)
Age in years: median (IQR)	86 (83–89)	87 (84–90)
Age at death		
65–74	294 (3.0)	254 (1.7)
75–84	4075 (41.3)	5028 (32.5)
85–94	5215 (52.9)	9232 (59.6)
95–104	277 (2.8)	958 (6.2)
≥ 105	1 (0.0)	11 (0.1)
Year of death		
2005	1204 (12.2)	1772 (11.4)
2006	2236 (22.7)	3199 (20.7)
2007	2351 (23.8)	3473 (22.4)
2008	2133 (21.6)	3619 (23.4)
2009	1938 (19.7)	3420 (22.1)
Age at cancer diagnosis: Median (IQR)	83 (78–86)	Not applicable
Years from diagnosis to death years: Median (IQR)	1.6 (0.2–5.6)	Not applicable
Location of residence (remoteness area)		
Major cities	6147 (62.3)	9530 (61.6)
Inner Regional	2777 (28.2)	4400 (28.4)
Outer Regional	872 (8.8)	1410 (9.1)
Remote	39 (0.4)	81 (0.5)
Very Remote	5 (0.1)	2 (0.0)
Missing	22 (0.2)	60 (0.4)
Socioeconomic disadvantage index		
(most disadvantaged) 1–2	1160 (11.8)	1862 (12.0)
3–4	2831 (28.7)	4470 (28.9)
5–6	2032 (20.6)	3085 (19.9)
7–8	1418 (14.4)	2248 (14.5)
(least disadvantaged) 9–10	2019 (20.5)	3183 (20.6)
Missing	402 (4.1)	635 (4.1)
Comorbidity burden^a^ (based on hospitalisations)		
0	3105 (31.5)	4451 (28.8)
1–2	1500 (15.2)	2068 (13.4)
≥ 3	1713 (17.4)	1578 (10.2)
Unable to calculate, no hospitalisations	3544 (35.9)	7386 (47.7)
Comorbidity burden^b^ (based on prescriptions)		
0	461 (4.7)	846 (5.5)
1–2	1298 (13.2)	2130 (13.8)
3–5	3865 (39.2)	6104 (39.4)
≥ 6	4238 (43.0)	6403 (41.4)
Living in residential aged care at any time during the last 6 months of life	3659 (37.1)	8940 (57.7)
Place of death		
Hospital	5740 (58.2)	6861 (44.3)
Residential aged care	2507 (25.4)	6435 (41.6)
Other	1615 (16.4)	2187 (14.1)

© Commonwealth of Australia 2017

^a^Charlson comorbidity index calculated using hospitalisations between 18 and 7 months before death

^b^Rx-Risk comorbidity index calculated using dispensing history in the 6 month period before the last 6 months of life (between month 12 and 7 before death)

### Resource use and costs in the last 6 months of life

On average, decedents in the cancer cohort were dispensed 41 medicines (vs. 38 medicines in the comparison cohort), received 90 clinician visits/procedures (vs. 66 in the comparison cohort) and had approximately three hospital admissions (vs. two in the comparison cohort). The mean number of ED visits was similar in both cohorts at about one visit/decedent in the last 6 months of life [Additional file 1: Table S1].

**Fig. 1:**
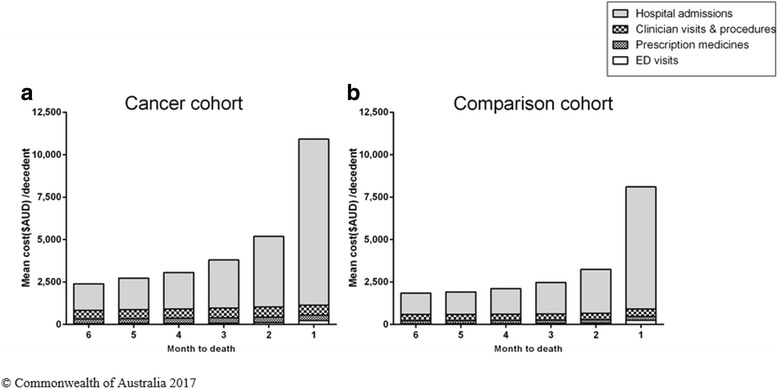
Total health care costs in the last six months of life, by month and by health service type for the (**a**) cancer cohort and (**b**) comparison cohort

The mean total cost associated with health care in the last 6 months of life was higher in the cancer cohort ($28,091 per decedent) than the comparison cohort ($19,696 per decedent). Costs were driven primarily by hospitalisations, accounting for about 80% of total costs in both cohorts [Fig. 1].

#### Prescription medicines

The mean cost of medicines per person in the cancer cohort was $1840 compared with $1234 for the comparison cohort. [Additional file 1: Table S1] The difference was driven by the costs of antineoplastic and immunomodulation agents (mean of $469 per person in the cancer cohort vs. $5 in the comparison cohort).

#### Clinician visits and procedures

Differences in utilisation can be attributed mainly to higher numbers of medical specialist visits and pathology services in the cancer cohort. The greatest difference in health service costs related to diagnostic procedures at a mean cost of $1041 per person in the cancer cohort compared to $568 for the comparison cohort. The cost of therapeutic procedures also differed substantially between cohorts, with mean costs of $877 and $464 per person in the cancer and comparison cohorts respectively [Additional file 1: Table S1].

#### Hospitalisations

The difference in hospitalisation costs was driven by the higher number of admissions in the cancer cohort (mean of three episodes per person costing $22,852 in total) than the comparison cohort (two episodes per person costing $15,893 in total). [Additional file 1: Table S1] Only 15% of the cancer cohort and 3% of the comparison cohort received a palliative service while in hospital; and 4% of the cancer cohort and <1% of the comparison cohort were admitted to a hospice [Additional file 1: Table S2].

#### Resource use and costs by month, during the last 6 months of life [Figs. 2 and 3]

Rates of health service use and associated costs increased over the last 6 months of life and peaked in the last month of life. Total costs for both cohorts and in each month to death are driven by the cost of hospitalisations [Fig. 1].

**Fig. 2:**
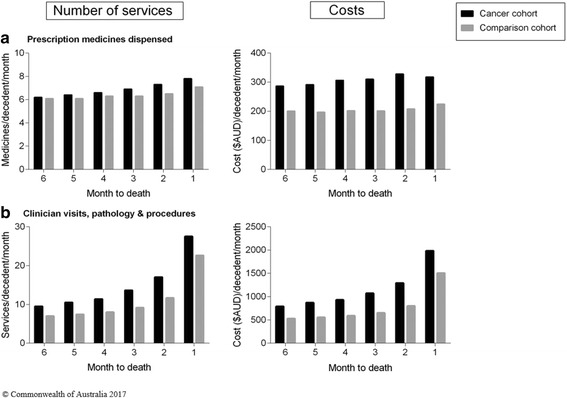
Health service use and associated costs in the last 6 months of life, month. **a** Prescription medicines dispensed; and (**b**) Clinician visits, pathology and procedures

**Fig. 3:**
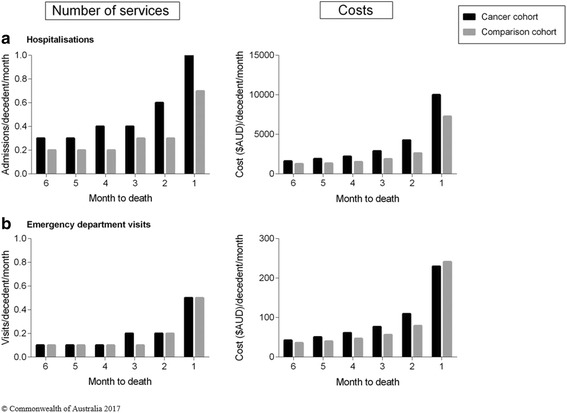
Health service use and associated costs in the last 6 months of life, by month. **a** Hospital admissions and (**b**) Emergency Department visits

#### Factors associated with resource use and costs

**Fig. 4:**
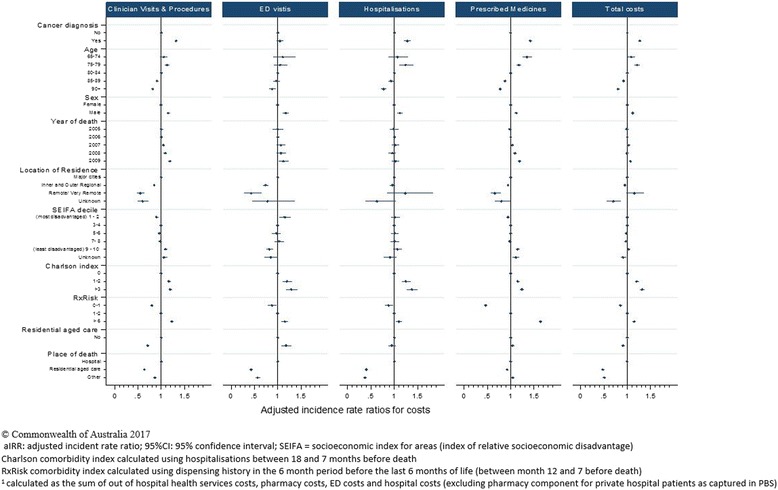
Multivariable analysis examining the associations between cohort characteristics and costs in the last 6 months of life, by health service type and total cost^1^

Multivariable analyses demonstrate that cancer decedents had significantly higher rates of prescription medicines (adjusted IRR: 1.09; 95% CI: 1.08–1.11, *p* < 0.001); clinician visits and procedures (adjusted IRR: 1.23; 95% CI: 1.20–1.25, *p* < 0.001); hospitalisations (adjusted IRR: 1.26; 95% CI: 1.23–1.30, *p* < 0.001) and ED visits (adjusted IRR: 1.05; 95% CI: 1.02–1.08, *p* < 0.001) than the comparison cohort. Decedents aged over 90 at death had lower rates of prescription medicines (adjusted IRR: 0.92; 95% CI: 0.90–0.93, *p* < 0.001); clinician visits and procedures (adjusted IRR: 0.81; 95% CI: 0.79–0.84, *p* < 0.001); hospitalisations (adjusted IRR: 0.76; 95% CI: 0.74–0.79, *p* < 0.001) and ED visits (adjusted IRR: 0.91; 95% CI: 0.88–0.95, *p* < 0.001) than younger decedents [Fig. 4].

**Fig. 5:**
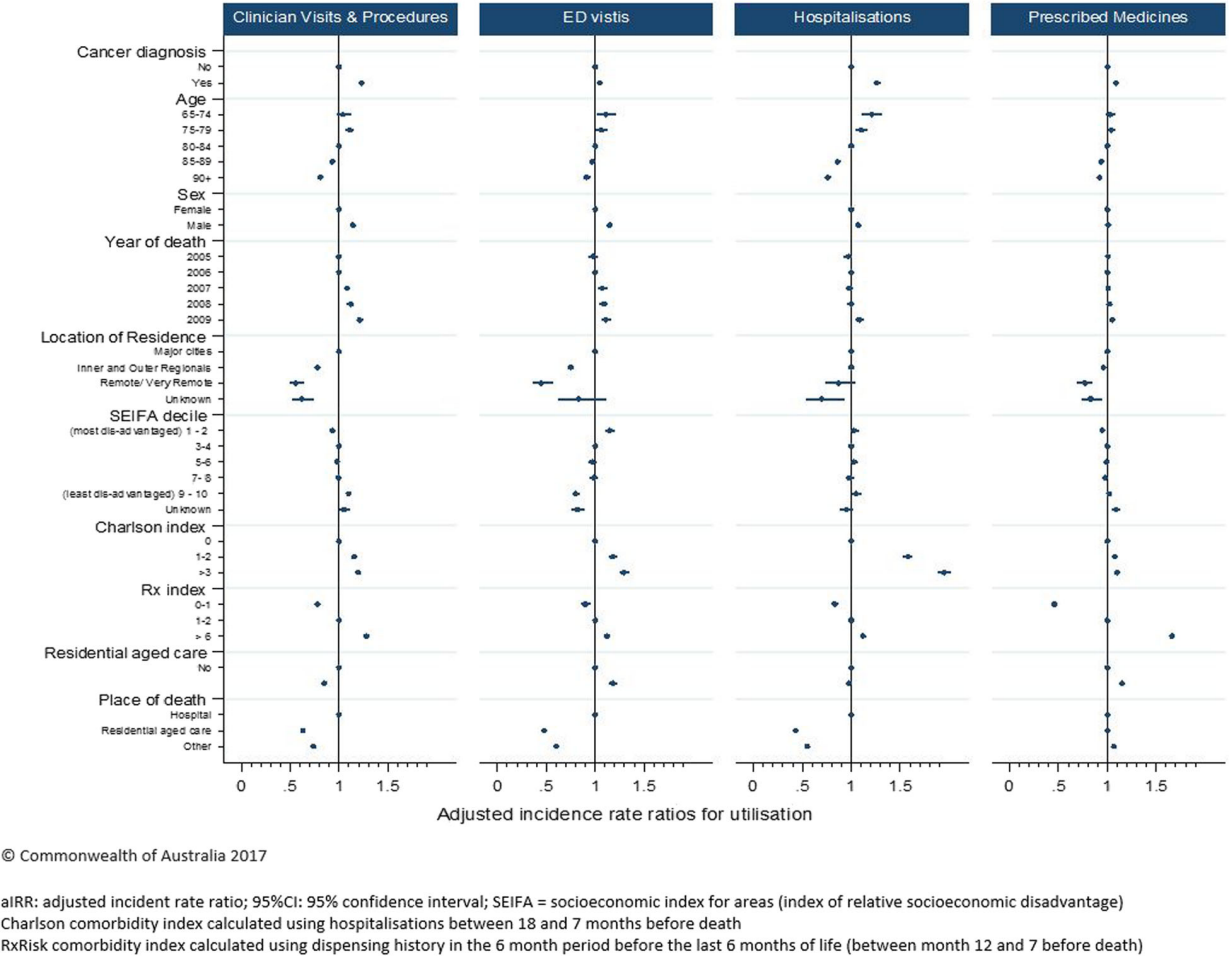
Multivariable analysis examining the associations between cohort characteristics and health service use in the last 6 months of life

Overall, cancer decedents incurred 27% higher health care costs than non-cancer decedents, (adjusted IRR: 1.27; 95% CI: 1.24–1.30, *p* < 0.001) [Fig. 5]. The cancer cohort had 42% higher costs for prescribed medicines (adjusted IRR: 1.42; 95% CI: 1.39–1.46, *p* < 0.001), 32% higher costs for clinician visits and procedures (adjusted IRR: 1.32; 95% CI: 1.29–1.35, *p* < 0.001) and 28% higher costs for hospitalisations (adjusted IRR: 1.28; 95% CI: 1.21–1.35, *p* < 0.001) than the comparison cohort. There was no significant difference in costs associated with ED visits (adjusted IRR: 1.05; 95% CI: 0.98–1.12, *p* = 0.166) [Fig. 5]. A number of factors were associated with lower health care costs: decedents aged over 90 at death had 20% lower health care costs than decedents aged 80–84 (adjusted IRR: 0.80; 95% CI: 0.77–0.82); decedents residing in residential aged facilities incurred 9% lower total costs (adjusted IRR: 0.91; 95% CI: 0.88–0.95); and those who died in residential aged care incurred less than half the costs of those who died in hospital (adjusted IRR: 0.47; 95% CI: 0.45–0.49) [Fig. 5].

## Discussion

This study used routine health care data collections to quantify and characterise factors associated with health care use and costs at the end of life in elderly decedents. Our results suggest wide variation in end-of-life care, particularly when comparing decedents with and without a cancer history. Decedents with a cancer history had higher rates health services use and higher associated costs than non-cancer decedents. Age at death was also a determinant of end-of-life care, with older decedents in both cohorts using fewer health care services than younger decedents. This is the first study of its kind in Australia, yet the results align with studies conducted internationally in terms of the impact of age on health services use and distribution of health care costs, with hospitals being the main driver of end-of-life costs [8].

Our results suggest systematic differences in the care received by elderly populations, particularly those aged 85 years and over. The patterns of care received by this group may suggest a different attitude towards active or intensive treatment for this group compared with their younger counterparts. Our finding that older decedents were less likely to use hospital and ED services are similar to another study examining the entire NSW population which reported that decedents aged 90 and older were 60% less likely to have more than three hospital episodes and 85% less likely to spend time in the ICU than those aged 60–79 years at death [29]. Our results show that not only were elderly decedents (both cancer and non-cancer) less likely to be admitted to hospital, but also received fewer clinician visits and prescription medicines at the end of life. The reduced services in elderly decedents may be compounded by the fact that this group are more likely to live in residential aged care settings; those who died in residential aged care incurred half the health care costs of those who died in hospital. While some literature suggests that lower rates of hospital and ED use at the end of life may represent quality care, [30] there is a need to further investigate whether the reductions in end-of-life health service use in elderly decedents translate into appropriate treatment that is consistent with patient preferences [31, 32]. We suggest that the differences in care observed in this and other studies does not necessarily represent inequities in treatment. Rather, the very elderly may have their end-of-life needs met with other sources of care [33, 34].

Services such as palliative hospital admissions and use of hospice care are regarded as important for delivering quality end-of-life care [7]. The proportion of people receiving palliative services in hospital in the last 6 months of life was low (14.7% in the cancer cohort and 3.1% for the comparison cohort); hospice admissions were also low (4.1% and 0.5% respectively, for the same cohorts).While our findings are consistent with other studies reporting the challenges of elderly patients accessing palliative services in acute care settings, [35] a limitation of our study is that rates of palliative care services delivered may be underestimated due to coding practices in Australia not being tied to hospital payment [36]. Nevertheless, the low rates of palliative services in this study are important from a health care planning perspective and warrant further examination. National data demonstrate that less than half of all patients who die in hospital receive any palliative care service [36].

Our findings have important policy implications within the broader social and economic context of end-of-life care. For instance, international studies and surveys of the Australian population indicate that most people with a terminal condition would prefer to die at home and prefer a symptom management approach rather than intensive treatment or heroic life-saving measures [37–39]. However, we found that hospital was the most common place of death and also placed the greatest burden on the health system in terms of costs. This indicates the need for more widespread discussion between patients, providers, caregivers, and health system managers about end-of-life planning (e.g., measures such as advanced care directives). Indeed, patients’ awareness that their condition is terminal and end-of-life discussions with clinicians have been demonstrated to result in the delivery of care in-line with patient preferences [31, 40]. There is also evidence that suggests there is merit in increasing resources for community-based palliative care services [41, 42]. While our study is comprehensive in that it captures nearly all health services from a health payer perspective, recent studies considering costs more broadly found that between one-fifth and one-third of overall end-of-life care costs can be attributed to informal care givers [43]. Costs of informal caregiving and patient preferences are not captured in routinely collected data holdings but this information is needed to fully understand how to maximise the value of end-of-life care services at a population-level in a health care environment with limited resources.

There are a number of strengths of our study, including our comprehensive patient-level analysis using multiple linked routine data collections. Our findings relating to DVA clients may not be generalisable to the elderly Australian cancer population. However, there is evidence that DVA clients have similar rates of health service use when compared with Australians of a similar age [44, 45] and comparison with recent studies on end-of-life hospital care in the general population suggest similar rates of hospital admissions and ED visits [29, 46].

A study limitation is that we do not provide information on patterns of end-of-life care in people under 65 years of age, a group that represents about 20% of all deaths in Australia [47] and up to one third of cancer deaths [20, 48]. Also there may have been changes in practice in Australia since our study period ended in 2009; however, the data reflect the most recent available at the time this research was undertaken. Another potential limitation of our study is the retrospective study design that does not necessarily reflect the challenges associated with predicting survival time in real world clinical practice [49]. However, some research comparing prospective and retrospective designs found similar patterns of end-of-life health care [13, 50].

## Conclusion

Decedents with a history of cancer used more services and incurred higher costs in the last 6 months of life than decedents without a cancer history. We also found evidence of a shift in treatment patterns by age with decedents aged 65–84 years receiving a greater number and range of services (particularly hospital based services) than those aged85 years and older. Future research is required to understand which patterns of care are associated with improved patient-reported care and to understand which patterns of care represent the best value in terms of appropriateness, quality and patient preferences.

## Additional file

Additional file 1: Table S1. Mean and median health service use and costs in the last 6 months of life, by health service type, and cohort. **Table S2.** Mean and median health service use and costs in the last 6 months of life, by health service type, and cohort. (DOCX 24 kb)

## Abbreviations

CT: Computed tomography
DVA: Department of Veterans Affairs
ED: Emergency Department
EOL-CC: End-of-life cancer care study
ICU: Intensive care unit
MRI: Magnetic resonance imaging
NSW: New South Wales
