# Supplementary material for: Health care use and costs at the end of life: a comparison of elderly Australian decedents with and without a cancer history
Source: BMC Palliat Care. 2017 Jun 21;17:1. doi: 10.1186/s12904-017-0213-0 (PMC5480123; doi:10.1186/s12904-017-0213-0)
Supplement: Additional file 1: Table S1. — Mean and median health service use and costs in the last 6 months of life, by health service type, and cohort. Table S2. Mean and median health service use and costs in the last 6 months of life, by health service type, and cohort. (DOCX 24 kb) [file 12904_2017_213_MOESM1_ESM.docx]

**Supplementary Table 1.** Mean and median health service use and costs in the last six months of life, by health service type, and cohort

|  | **Resource utilisation** | | **Cost (AUD)** | |
| --- | --- | --- | --- | --- |
|  | **Cancer cohort**  **(N = 9862)** | **Comparison cohort**  **(N = 15,483)** | **Cancer cohort**  **(N = 9862)** | **Comparison cohort**  **(N = 15,483)** |
| **Overall costs^1^** | Not applicable | |  |  |
|  |  |  |  |  |
| Mean (95% CI) |  |  | 28,091  (27,510-28,671) | 19,696  (19,230-20,162)) |
| Median (IQR) |  |  | 21,265  (11,006-37,235) | 12,122  (3637-26,231) |
| **Prescription medicines dispensed** |  |  |  |  |
| ≥1 medicine dispensed, n (%) | 9696 (98.3) | 15,013 (97.0) |  |  |
| Mean (95%CI) | 41.3 (40.8-41.8) | 38.4 (38.0-38.8) | 1840 (1795-1885) | 1234 (1213-1254) |
| Median (IQR) | 38 (24-55) | 35 (21-52) | 1242 (659-2228) | 993 (522-1623) |
| **All clinical consultations, pathology and procedures** |  |  |  |  |
| ≥1 clinical consultation, pathology or procedure, n (%) | 9850 (99.9) | 15,378 (99.3) |  |  |
| Mean (95%CI) | 89.5 (88.2-90.8) | 65.7 (64.8-66.7) | 6938 (6828-7048) | 4639 (4560-4718) |
| Median (IQR) | 74 (141-119) | 44 (21-90) | 5799 (3097-9285) | 3059 (1271-6316) |
| *Clinical consultations, pathology and procedures - Out of Hospital Only (OOH)* |  |  |  |  |
| ≥1 clinical consultation, pathology or procedure (Out of Hospital), n (%) | 9774 (99.1) | 15,137 (97.8) |  |  |
| Mean (95%CI) | 36.5 (35.9-37.1) | 26.8 (26.4-27.3) | 3352 (3287-3416) | 2233 (2192-2275) |
| Median (IQR) | 30 (18-47) | 21 (12-34) | 2493 (1300-4389) | 1432 (761-2811) |
| **Hospital admissions** |  |  |  |  |
| *Episodes* |  |  |  |  |
| ≥1 hospital admission, n (%) | 8494 (91.2) | 10,899 (70.4) |  |  |
| Mean (95%CI) | 2.9 (2.9-3.0) | 2.0 (2.0 -2.0) | 22,852  (22,230-23,474) | 15,893  (15,431-16,355) |
| Median (IQR) | 2 (1-4) | 1 (0-3) | 15,437  (5491-31,334) | 8218 (0-21,082) |
| *Days accrued in hospital* |  |  |  |  |
| Mean (95%CI) | 27.0 (26.4 – 27.5) | 19.5 (19.0 – 20.0) | Not applicable | |
| Median (IQR) | 19 (5-39) | 8 (0-26) |  |  |
| *Pharmacy adjusted* |  |  |  |  |
| Mean (95%CI) | Not applicable | | 22,328  (21,755-22901) | 15,729  (15,271-16,187) |
| Median (IQR) |  |  | 15,186  (5411-30,538) | 8120 (0-20,920) |
| **Emergency department visits** |  |  |  |  |
| ≥1 ED visit, n (%) | 6360 (64.5) | 9141 (59.0) |  |  |
| Mean (95%CI) | 1.3 (1.2-1.3) | 1.1 (1.1-1.1) | 571 (558-583) | 500 (491-510) |
| Median (IQR) | 1 (0-2) | 1 (0-2) | 445 (0 -890) | 445 (0-805) |

n: Number of decedents with at least one service

^1^ Overall costs = sum of prescribed medicines, clinical consultations, pathology and procedures (out of hospital only), pharmacy adjusted hospitalisations and ED visits.

**Supplementary table 2.** Mean and median health service use and costs in the last six months of life, by health service type, and cohort

|  | **Resource utilisation** | | **Cost (AUD)** | |
| --- | --- | --- | --- | --- |
|  | **Cancer cohort**  **(N = 9862)** | **Comparison Cohort**  **(N = 15,483)** | **Cancer cohort**  **(N = 9862)** | **Comparison Cohort**  **(N = 15,483)** |
| **Clinical consultations, pathology and procedures, median (IQR)** | 74 (41 - 119) | 44 (21 - 90) | Not applicable | |
| *Clinical consultations* |  |  |  |  |
| General practitioners |  |  |  |  |
| Mean (95% CI) | 14.2 (13.9 – 14.4) | 12.0 (11.8 – 12.1) | 912 (896-928) | 757 (747-768) |
| Median (IQR) | 11 (7 - 18) | 10 (5 - 15) | 700 (398-1167) | 588 (319-977) |
| Specialists |  |  |  |  |
| Mean (95% CI) | 9.8 (9.5 – 10.0) | 6.5 (6.4 – 6.7) | 1029  (1004-1055) | 719 (700-738) |
| Median (IQR) | 6 (2 - 13) | 2 (0 - 8) | 607 (165-1418) | 222 (0-925) |
| Other medical practitioners |  |  |  |  |
| Mean (95% CI) | 0.8 (0.7 – 0.8) | 0.6 (0.6 – 0.7) | 84 (81-87) | 76 (74-79) |
| Median (IQR) | 0 (0 – 0) | 0 (0 – 0) | 0 (0-125) | 0 (0-112) |
| Pain and palliative |  |  |  |  |
| Mean (95% CI) | 0.4 (0.4 – 0.5) | 0 (0 – 0) | 50 (45-56) | 4 (3-5) |
| Median (IQR) | 0 (0 – 0) | 0 (0 – 0) | 0 (0-0) | 0 (0-0) |
| Other health care professionals (dentists, nurses, allied & alternative health practitioners) |  |  |  |  |
| Mean (95% CI) | 6.9 (6.7 – 7.0) | 5.4 (5.3 – 5.6) | 1232  (1191-1273) | 901 (870-932) |
| Median (IQR) | 4 (1-9) | 2 (0-7) | 331 (60-1680) | 120 (0-814) |
| *Pathology* |  |  |  |  |
| Collection |  |  |  |  |
| Mean (95% CI) | 9.1 (8.9 – 9.3) | 6.8 (6.7 – 7.0) | 97 (94-100) | 70 (69-72) |
| Median (IQR) | 5 (2 - 12) | 3 (1 - 9) | 52 (18-124) | 27 (3-80) |
| Number of specific tests |  |  |  |  |
| Mean (95% CI) | 33.8 (33.1 – 34.6) | 25.5 (24.9 – 26.0) | 924 (904-945) | 635 (621-649) |
| Median (IQR) | 23 (9 - 46) | 13 (3 - 34) | 617 (244 - 1241) | 312 (78 - 842) |
| *Procedures* |  |  |  |  |
| Surgery |  |  |  |  |
| Mean (95% CI) | 1.4 (1.3 – 1.4) | 0.7 (0.7 – 0.7) | 524 (504-544) | 309 (296-322) |
| Median (IQR) | 1 (0 - 2) | 0 (0 - 1) | 36 (0 - 622) | 0 (0-162) |
| Therapeutic (eg radiotherapy, nuclear medicine) |  |  |  |  |
| Mean (95% CI) | 5.4 (5.2 – 5.7) | 2.7 (2.5 – 2.8) | 877 (837-917) | 464 (439-489) |
| Median (IQR) | 1 (0 - 7) | 0 (0 - 2) | 99 (0 - 898) | 0 (0-281) |
| Diagnostic (eg ultrasound, CT scan) |  |  |  |  |
| Mean (95% CI) | 6.9 (6.7 – 7.0) | 4.8 (4.7 – 4.9) | 1041  (1019-1063) | 568 (554-582) |
| Median (IQR) | 5 (2 - 10) | 3 (0 - 7) | 740 (189-1525) | 234 (0-772) |
| **All hospital admissions, n (%)** | 8494 (91.2) | 10,899 (70.4) | Not applicable | |
| *Hospice admissions, n (%)* | 401 (4.1) | 81 (0.5) |  |  |
| *Admitted for a palliative service, n (%)* | 1451 (14.7) | 486 (3.1) |  |  |
| *All other hospitalisations, n (%)* | 8259 (83.7) | 10,861 (70.1) |  |  |
| ICU, n (%) | 703 (7.1) | 1051 (6.8) |  |  |
| **Emergency department visits** |  |  |  |  |
| *ED visits without subsequent hospital admission, n (%)* | 1901 (19.3) | 2451 (15.8) | Not applicable | |
| Mean (95% CI) | 0.3 (0.3 – 0.7) | 0.2 (0.2 – 0.2) | 90 (86-94) | 72 (69-75) |
| Median (IQR) | 0 (0-0) | 0 (0-0) | 0 (0-0) | 0 (0-0) |
| *ED visits with subsequent hospital admission, n (%)* | 5860 (59.4) | 8354 (54.0) | Not applicable | |
| Mean (95% CI) | 1.0 (1.0 – 1.0) | 0.9 (0.8 – 0.9) | 481 (470-492) | 428 (420-437) |
| Median (IQR) | 1 (0-2) | 1 (0-1) | 445 (0-890) | 445 (0-684) |
